# Possible Mechanisms by Which Enzymatic Degradation of Human Serum Albumin Can Lead to Bioactive Peptides and Biomarkers

**DOI:** 10.3389/fmolb.2018.00063

**Published:** 2018-07-09

**Authors:** Ulrich Kragh-Hansen

**Affiliations:** Department of Biomedicine, University of Aarhus, Aarhus, Denmark

**Keywords:** albumin, fragments, bioactive peptides, biomarkers, FcRn receptor, endosome, enzymatic degradation, lysosome

## Abstract

Partial enzymatic degradation of human serum albumin *in vivo* can lead to the generation of peptides with novel functions or to peptides that might serve as biomarkers for disease. In pathological conditions, biomarkers are possibly produced from the protein in the lysosomes and set free by cell death, or cell death could release acid endoproteases which produce biomarkers by degrading extracellular albumin. Alternatively, lysosomes or secretory granules can be stimulated to release enzymes which produce bioactive peptides from albumin. In physiological conditions, it is proposed that bioactive peptides can be made by enzymatic attack on the protein bound to the endosomal neonatal Fc receptor. The peptides formed could leave the cell, together with native albumin, by exocytosis. Thus, the receptor could have a new function in addition to saving albumin from degradation in the lysosomes. Large amounts of albumin are degraded every day, and this fact can compensate for the short *in vivo* half-lives of the bioactive peptides. One or more of the procedures outlined above could also apply to other plasma proteins or to structural proteins.

## Introduction

More than 7,000 naturally occurring peptides have been identified (Fosgerau and Hoffmann, 2015). Endogenous peptides can be produced in three major ways (Dave et al., 2016). Some of the peptides are encoded directly from small open reading frames and are released directly into the cytoplasm. Another type is biosynthetically-derived peptides which are encoded within inactive precursor proteins and set free after proteolysis. Finally, bioactive peptides with novel functions and biomarkers for disease can be made, when native, functioning proteins are degraded. For obtaining useful amounts of the latter two types of peptides, which are known as crypteins or cryptides (Dave et al., 2016; Iavarone et al., 2018), it is advantageous that the “mother protein” is abundant and has a pronounced turn-over. By useful amounts of bioactive peptides is meant an amount sufficient to give a physiological response, and in the case of biomarkers it means detectable amounts.

Human serum albumin (HSA) must be a good candidate as such a “mother protein.” It is synthesized in the liver, and in healthy adults it is continuously secreted and degraded at a rate of ca. 14 g per day (Peters, 1996; Kragh-Hansen, 2016). The total amount of the protein in the body of a 70-kg subject is ca. 360 g, of which about two-thirds are in extravascular spaces and about one-third is within the bloodstream. By contrast to its synthesis, its sites of degradation, and thereby possible production of peptides, are widespread. Most of the protein is hydrolyzed in muscles and skin, but several other types of cells and organs are involved in its catabolism (Peters, 1996).

In accordance with the proposal of HSA as a “mother protein” several bioactive peptides and biomarkers originating from the protein have been found in different body fluids. As apparent from Table 1, peptides have been found in bronchoalveolar lavage fluid, bile, urine and blood; the latter in the form of plasma or haemofiltrate. The examples given in Table 1 are a minimum, because, for example, Raida et al. (1999) detected 28 fragments in haemofiltrate, Lankisch et al. (2011) found and sequenced 16 peptides in bile, and Theodorescu et al. (2005) sequenced 20 peptides found in urine. In addition, Stoop et al. (2010) identified 63 different peptides isolated from human cerebrospinal fluid.

**Table 1:** Bioactive peptides and biomarkers derived from human serum albumin.

**No.^a^**	**Sequence in HSA**	**Amino acid sequence**	**Name**	**Source^b^**	**Function**	**References**
1	1–18	DAHKSEVAHRFKDLGEEN		Urine^c^	Biomarker for renal disease	Chalmers et al., 2005
2	1–18	DAHKSEVAHRFKDLGEEN		Urine	Biomarker for acute kidney injury	Metzger et al., 2010^d^
3	1–18	DAHKSEVAHRFKDLGEEN		Urine	Biomarker for chronic kidney disease	Good et al., 2010^d^
4	1–19	DAHKSEVAHRFKDLGEENF		Blood		Machtejevas et al., 2004
5	1–19	DAHKSEVAHRFKDLGEENF		Urine	Predictor for graft-vs. -host disease after hematopoietic stem cell transplantation	Weissinger et al., 2014
6	1–20 (21)(23)	DAHKSEVAHRFKDLGEENFK (A)(LV)		Blood		Machtejevas et al., 2004
7	1–21	DAHKSEVAHRFKDLGEENFKA		BALF^e^	BPD^e^	Vento et al., 2009
8	1–21	DAHKSEVAHRFKDLGEENFKA		Blood	Copper binding site	Richter et al., 1999
9	1–21 (22)(23)	DAHKSEVAHRFKDLGEENFKA (L)(V)		Urine	Biomarker for diabetic nephropathy	Alkhalaf et al., 2010
10	1–24 (26)	DAHKSEVAHRFKDLGEENFKA LVL(IA)		Blood		Kausler and Spiteller, 1991
11	25–30	IAFAQY		Blood		Kausler and Spiteller, 1991
12	65–70	SLHTLF	Cabin-A1	HSA	Inhibitor of Cathepsin B	Nakagomi et al., 2002
13	115–186	LVRP…….DELR		HSA	Insulin stimulation and activation of pyruvate dehydrogenase	Ueno et al., 1986, 1987
14	138–144	YLYEIAR	Acein-1	Plasma	ACE-inhibitor^f^	Nakagomi et al., 1998
15	142–149	IARRHPYF	HRP	Plasma	Histamine release from mast cells and activation of leukocytes	Carraway et al., 1989; Cochrane et al., 2003
16	149–159	FYAPELLFFAK		Bile	Biomarker for bile duct diseases	Lankisch et al., 2011
17	213–214	AW		HSA	ACE-inhibitor	Nakagomi et al., 2000
18	210–218	AFKAWAVAR	Albutensin A	HSA	ACE-inhibitor	Nakagomi et al., 2000
19	210–218	AFKAWAVAR	Albutensin A	HSA	Inhibitor of Cathepsin B	Nakagomi et al., 2002
20	330–333	FLYE		Blood		Kausler and Spiteller, 1991
21	399–406	GEYKFQNA		BALF^e^	BPD^e^	Vento et al., 2009
22	403–407	FQNAL	Cabin-A2	HSA	Inhibitor of Cathepsin B	Nakagomi et al., 2002
23	407–423 (425)	LLVRYTKKVPQVSTPTL(VE)		Blood		Machtejevas et al., 2004
24	(407)(408) 409–423	(L)(L)VRYTKKVPQVSTPTL		Blood	Activate the MRGPRX2 receptor in mast cells	Karhu et al., 2017
25	408–423	LVRYTKKVPQVSTPTL		Urine	Biomarker for renal disease	Chalmers et al., 2005
26	408–423	LVRYTKKVPQVSTPTL		Blood	Biomarker for renal disease	Mohr et al., 2015
27	408–423	LVRYTKKVPQVSTPTL		Urine	Predictor for graft-vs. -host disease after hematopoietic stem cell transplantation	Kaiser et al., 2004
28	408–423	LVRYTKKVPQVSTPTL	EPI-X4	Blood	Interacts with the CXCR4-receptor	Zirafi et al., 2015, 2016
29	408–423	LVRYTKKVPQVSTPTL		Urine	Biomarker for prostate cancer	Theodorescu et al., 2005; Mohr et al., 2015
30	408–425	LVRYTKKVPQVSTPTLVE		Urine	Biomarker for IgA nephropathy	Haubitz et al., 2005
31	409–423	VRYTKKVPQVSTPTL	P-1	HSA	Histamine release from mast cells	Sugiyama et al., 1989
32	409–423	VRYTKKVPQVSTPTL		Blood		Machtejevas et al., 2004
33	497–506	YVPKEFNAET		Urine	Biomarker for major depressive disorder	Wang et al., 2014

a *The fragments are numbered from the N-terminal end of HSA*.

b *Isolated from blood in the form of plasma (no. 22) or haemofiltrate (no. 4, 6, 7, 9, 10, 19, 21, 24, 26, 30), from urine (no. 1, 2, 3, 5, 8, 23, 25, 27, 28, 31), or from bile (no. 15), or obtained by tryptic/chymotryptic hydrolysis of HSA (no. 11, 12, 16, 17, 18, 20) or plasma (no. 13) or by peptic hydrolysis of HSA (no. 29) or plasma (no. 14)*.

c *The majority of the peptides found in urine are plasma peptides excreted in the urine (Kaiser et al., 2004; Candiano et al., 2008; Hortin and Sviridov, 2008; Alkhalaf et al., 2010; Wang et al., 2014)*.

d *The authors detected a total of 5 different N-terminal fragments (Metzger et al., 2010) and 9 fragments mostly from the N-terminal end (Good et al., 2010)*.

e *The fragments are found in bronchoalveolar lavage fluid (BALF), and their concentrations are increased in patients with bronchopulmonary dysplasia (BPD)*.

f *Inhibitor of angiotensin-I-converting enzyme*.

Where in the human body are the peptides produced? Yang et al. (2015) observed that plasma proteases did not degrade HSA in blood plasma or serum at pH 7.5. However, the fragments 1–24 and 1–26 appeared when dropping the pH to 6. Likewise, Ahnoff et al. (2015) found that acidification of plasma to pH 3 resulted in the appearance of HSA-fragment 402–407, whereas Zirafi et al. (2015) found that acidification to pH 4 resulted in the production of fragment 408–423. Furthermore, fragments 142–149 and 409–423 were isolated from peptic hydrolysates of human plasma and HSA, respectively, obtained at acidic pH (Table 1). These examples suggest that HSA-derived peptides are not produced at physiological pH. The above findings propose that they can be made locally in the body in very acidic milieus. A well-suited place for their formation could be intracellularly in acidic compartments such as endosomes and/or lysosomes (Zirafi et al., 2015, 2016).

## Production of peptides in pathological conditions

As described in the following, it seems relevant to suggest that the mechanisms by which HSA-derived peptides are made in pathological conditions differ from that by which the peptides are produced in physiological conditions. In the former type of conditions the mechanisms could be the following. (i) It is known that cellular uptake of HSA by pinocytosis or receptor-mediated endocytosis is followed by transfer of conformationally modified protein and of native protein in surplus to the lysosomes (Bern et al., 2015; Sand et al., 2015; Nilsen et al., 2016); see below. In that compartment, with a pH of about 4, HSA could be partially degraded by acid endoproteases like cathepsins and cysteine proteases. The peptides formed could be released into the extracellular spaces after cell death (Zirafi et al., 2015, 2016) (apoptosis, pyroptosis or necrosis). (ii) Alternatively or supplementary, proteases released as a result of cell death can fragmentate extracellular HSA (Yang et al., 2015). However, large amounts of peptide is probably not formed in this way, because although a local acidification is expected, the pH of the milieu will still be much higher than that of intact lysosomes in which the proteases are fully active. Most likely, the peptides produced by these two events serve as biomarkers (Table 1). (iii) Malignant tumors, inflammation and infection result in local extracellular acidification and secretion of proteases by lysosomal exocytosis or via specialized secretory granules from, e.g., immune cells (Zirafi et al., 2015, 2016). Thus, in this case the appearance of lysosomal proteases in the extracellular space is not the result of cell death. The HSA-fragments produced in such situations are usually peptides with biological effects. For example, the fragment known as EPI-X4 (Table 1) can be made in this manner, and when formed it has several biological effects through interaction with the CXCR4-receptor (Zirafi et al., 2015, 2016).

## Production of peptides in physiological conditions

**Figure 1:**
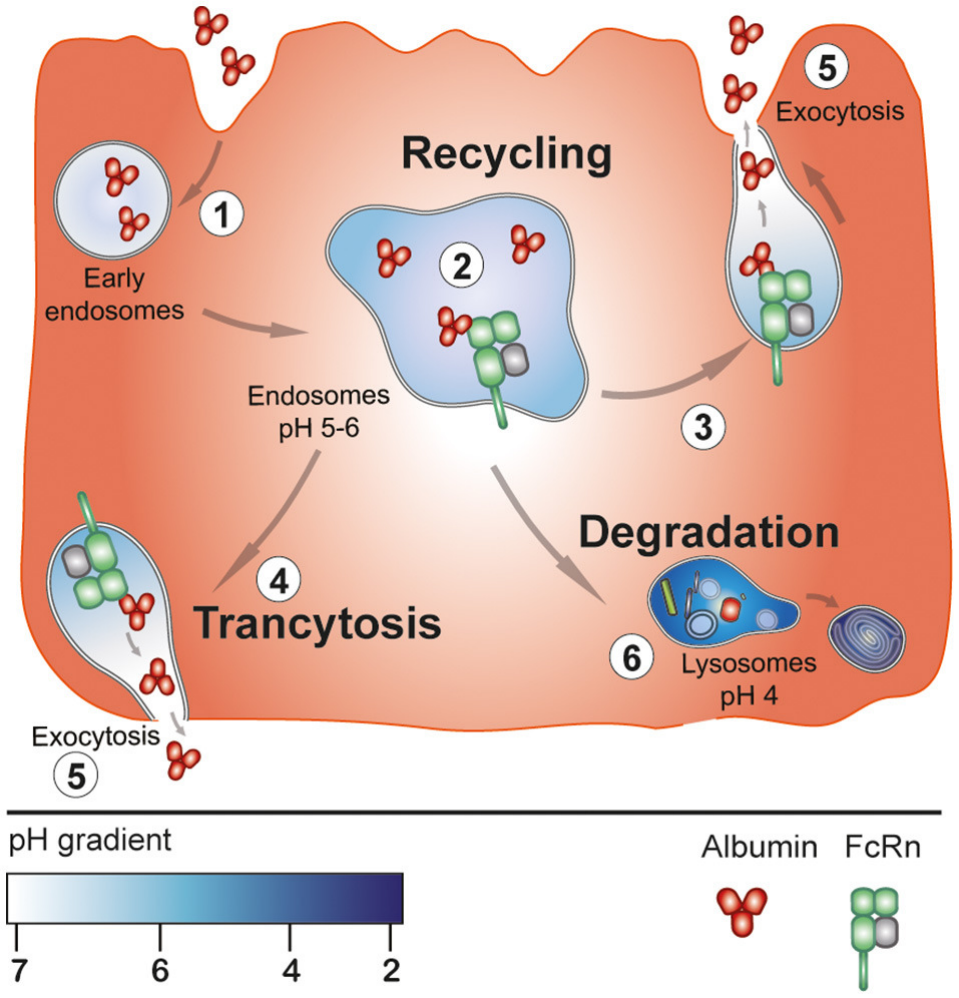
pH-dependent, FcRn-mediated cellular transport of HSA. Initially, HSA is taken up by the cell by pinocytosis (or receptor-mediated). After uptake the protein enters early endosomes (step 1). From there it is transferred to acidified endosomes having FcRn in the membrane. At this pH of 5-6, the protein binds strongly to the receptor at a 1:1 stoichiometry (step 2). HSA which does not bind to FcRn goes to degradation in the lysosomes (step 6). While bound to FcRn in the endosomes HSA is exposed to acid endoproteases the function of which is proposed to lead to the formation of bioactive peptides and other fragments. Intact protein and the fragments could leave the cell either in the form of recycling (steps 3 and 5) or by transcytosis (steps 4 and 5). In both cases protein leaves the receptor, because at physiological pH the interaction between the two components is very week. The illustration is inspired by figure 2 in Bern et al. (2015).

In physiological conditions bioactive peptides are not expected to be formed in connection with cell death or lysosomal exocytosis. Rather, fragments of HSA could be produced by a very different mechanism, namely one in which the intracellular, endosomal neonatal Fc receptor (FcRn) plays an essential role. FcRn is present in multiple cell types and tissues and is known as being most important for the half-life of HSA, because it protects HSA from degradation in the lysosomes (Bern et al., 2015; Sand et al., 2015; Nilsen et al., 2016). Actually, the receptor rescues as much HSA as the liver produces. The process is initiated by pinocytosis or receptor-mediated uptake of HSA (Figure 1). After uptake, the protein enters early endosomes. From there HSA is transferred to acidified endosomes, where FcRn predominantly resides, and at that pH (5-6) HSA binds strongly to the transmembrane receptor. Protein modified to such an extent that it has no affinity for FcRn and surplus protein will be destined for lysosomal degradation. Normally, endosomes with FcRn-bound HSA will fuse with the cell membrane. This event results in exocytosis in the form of recycling or transcytose of the protein, because HSA does not bind to FcRn at physiological pH (Figure 1) (Bern et al., 2015; Sand et al., 2015; Nilsen et al., 2016).

**Figure 2:**
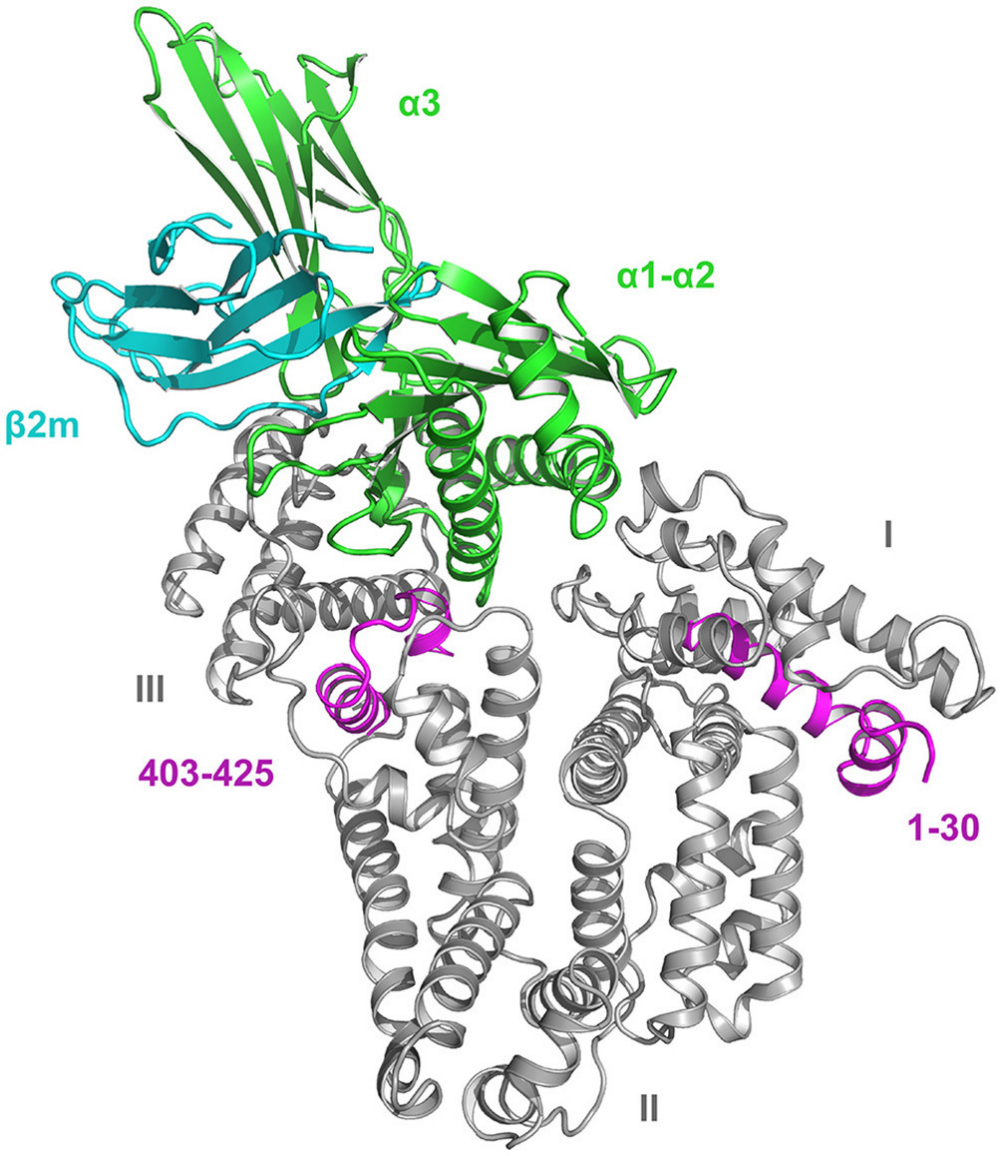
Crystal structure of the HSA-FcRn complex (Oganesyan et al., 2014). The domains (I-III) of HSA (66.5 kDa) are indicated. FcRn is composed of a long α-chain of 44 kDa (domains α1-α3) and a short β2-microglobulin unit of 12 kDa (β2m). Of these structures, only α3 has a transmembrane fragment and a cytosolic part. Domain III of HSA plays the essential role for binding, but domain I is also necessary. The sections in domain I and III marked in magenta represent the sequences 1–30 and 403–425, respectively. The figure was made with PyMol on the basis of the atomic coordinates (PDB ID: 4N0F) available at the RCSB Protein Data Bank.

During its stay in the endosomes, the majority of HSA is strongly bound to its single site on FcRn. Domain III of HSA is crucial for the pH-dependent binding (Figure 2), and the most important amino acids for the interaction are nine amino acids in the sequence 422–551 (Thr422, Val426, Leu460, Leu463, His464, Thr467, Phe507, Phe509, and Phe551) which mainly make hydrophobic interactions with residues of FcRn (Nilsen et al., 2016). Arg81, Asp89, Asn109, and Asn111 in domain I are not crucial for the complex formation but they modulate and stabilize the interaction (Nilsen et al., 2016). The parts of HSA which are not strongly involved in FcRn binding should be accessible to the proteases and thereby give rise to bioactive peptides. As apparent from Table 1 this is indeed the case, because all the fragments (except peptide no. 33) originate from the unbound parts of HSA. For illustrative examples, see the sections 1–30 and 403–425 (Figure 2) from which several peptides originate.

The explanation of the production of the many and diverse peptides could be a relative low selectivity of the acid endoproteases (Sun et al., 2013). Alternatively, different cell types could have different proteases. Finally, the type and quantity of the peptides produced could be modified, if the cell enters a state different from the “resting one,” for example by initiating an anti-inflammatory or anti-cancer response. The proposal of low selectivity and/or different proteases is supported by the observation that the peptides formed have very different N- and C-termini (Table 1).

The peptides formed are proposed to leave the cells by exocytosis along with intact, native HSA (Figure 1). What happens to the rest of HSA after the bioactive peptides have been cut out? The remaining parts could dissociate from FcRn and then be transferred to the lysosomes for complete degradation. However, evidence suggests that also the left-overs can leave the cells by exocytosis. Thus, large fragments have been found in both plasma and urine. Kshirsagar et al. (1984) found fragments with molecular weights of 19, 28 and 45 kDa in plasma, and Richter et al. (1999) identified the fragment 309–585 in the same medium. Chalmers et al. (2005) detected the fragments 454–491 and 507–585 in urine. Finally, Hortin and Sviridov (2008) reported the presence of 13 fragments (159–492 amino acids long) in both plasma and urine.

An advantage by the proposed mechanism is that the prerequisites exist in almost all cell types in the body. The bioactive peptides formed and their functions depend on the type of producing cell. However, peptides have (very) short *in vivo* half-lives, but this can be compensated for by the fact that large amounts of HSA are continuously degraded every day.

## Concluding remarks

Thus, the abundant HSA can act as a “mother molecule” and give rise to many different peptides. In pathological conditions, three ways of producing bioactive peptides or biomarkers seem to exist, which involve cell death or exocytosis from lysosomes or secretory granules. In physiological conditions, it is proposed that bioactive peptides are formed as a consequence of partial degradation of FcRn-bound HSA by acid endoproteases in the endosomes. The peptides formed, and perhaps also the remaining parts of HSA, leave the cell by exocytosis. Even though peptides have short half-lives *in vivo*, significant function can be performed, because large amounts of HSA are degraded every day.

One or more of the procedures described could also apply to other plasma proteins like apolipoproteins, fibrinogen and alpha-1-antitrypsin or to structural proteins like collagen. For example, IgG also binds reversibly to endosomal FcRn (Bern et al., 2015; Sand et al., 2015; Nilsen et al., 2016).

## Author contributions

UK-H made the literature research, worked out the proposals, and wrote the manuscript.

### Conflict of interest statement

The author declares that the research was conducted in the absence of any commercial or financial relationships that could be construed as a potential conflict of interest.
